# Delineating neural responses and functional connectivity changes during vestibular and nociceptive stimulation reveal the uniqueness of cortical vestibular processing

**DOI:** 10.1007/s00429-021-02394-6

**Published:** 2021-10-05

**Authors:** Judita Huber, Maxine Ruehl, Virginia Flanagin, Peter zu Eulenburg

**Affiliations:** 1 Graduate School of Systemic Neurosciences, Department of Biology II and Neurobiology, Ludwig-Maximilians-University, Munich, Germany; 2 Department of Neurology, University Hospital Munich, Ludwig-Maximilians-University, Munich, Germany; 3 German Center for Vertigo and Balance Disorders, University Hospital Munich, Ludwig-Maximilians-University, Munich, Germany; 4 Institute for Neuroradiology, University Hospital Munich, Ludwig-Maximilians-University, Munich, Germany

**Keywords:** Vestibular processing, Nociception, Functional connectivity, Neuroimaging

## Abstract

Vestibular information is ubiquitous and often processed jointly with visual, somatosensory and proprioceptive information. Among the cortical brain regions associated with human vestibular processing, area OP2 in the parietal operculum has been proposed as vestibular core region. However, delineating responses uniquely to vestibular stimulation in this region using neuroimaging is challenging for several reasons: First, the parietal operculum is a cytoarchitectonically heterogeneous region responding to multisensory stimulation. Second, artificial vestibular stimulation evokes confounding somatosensory and nociceptive responses blurring responses contributing to vestibular perception. Furthermore, immediate effects of vestibular stimulation on the organization of functional networks have not been investigated in detail yet. Using high resolution neuroimaging in a task-based and functional connectivity approach, we compared two equally salient stimuli—unilateral galvanic vestibular (GVS) and galvanic nociceptive stimulation (GNS)—to disentangle the processing of both modalities in the parietal operculum and characterize their effects on functional network architecture. GNS and GVS gave joint responses in area OP1, 3, 4, and the anterior and middle insula, but not in area OP2. GVS gave stronger responses in the parietal operculum just adjacent to OP3 and OP4, whereas GNS evoked stronger responses in area OP1, 3 and 4. Our results underline the importance of considering this common pathway when interpreting vestibular neuroimaging experiments and underpin the role of area OP2 in central vestibular processing. Global network changes were found during GNS, but not during GVS. This lack of network reconfiguration despite the saliency of GVS may reflect the continuous processing of vestibular information in the awake human.

**Supplementary Information:**

The online version contains supplementary material available at 10.1007/s00429-021-02394-6.

## Introduction

The vestibular system monitors active and passive head movements in all translational and rotational directions while at the same time sensing gravity. The interaction of human brain areas that compute this information from vestibular input is still not fully understood. Several notable aspects about the vestibular sense contribute to the complexity of human vestibular research. Under normal circumstances, vestibular information is accompanied by separate congruent sensory information such as vision or proprioception, and low-frequency vestibular processing in daily life activities does not seem to involve conscious awareness in healthy subjects. In functional neuroimaging studies, data acquisition under natural vestibular stimulation is not feasible yet, which further complicates the ongoing debate of the delineation of ‘pure’ vestibular responses and the localization of a vestibular network in humans. Thus, our understanding of the vestibular system in humans is still primarily based on single-unit recordings during real movement in non-human primates implicating a distributed set of cortical brain regions for processing different types of vestibular information. Vestibular information is transmitted from the periphery to the cortex via posterior thalamic vestibular nuclei to the somatosensory cortex and to the parieto-insular vestibular cortex (PIVC) located in the lateral sulcus adjacent to the insula. In this area, primate studies localized the primary vestibular cortex taking into account the large amount of neurons responding to vestibular input (Guldin and Grüsser 1998) even in the absence of visual input in darkness (Chen et al. 2010).

Neuroimaging studies using artificial vestibular stimulation like galvanic vestibular stimulation (GVS) suggested area OP2 in the parietal operculum as the human correlate of the PIVC (zu Eulenburg et al. 2012). GVS is a robust method to stimulate primary vestibular afferents and elicit motion perception without actual head-movement via small currents using electrodes attached to the mastoid (Kwan et al. 2019). However, it also may evoke somatosensory and nociceptive side-effects (Stephan et al. 2005; Lobel et al. 1998; Smith et al. 2012), which in particular have to be accounted for when regarding response in the parietal operculum, as it is a multisensory area responding to somatosensory and nociceptive stimulation (Horing et al. 2019; Eickhoff et al. 2007), and OP2 is located just adjacent to the secondary somatosensory area OP1. So far, one study implied a somatosensory control stimulus (*n* = 9), but did not describe responses in the parietal operculum and did not compare unilateral stimulations (Smith et al. 2012). Another difficulty common to most human neuroimaging studies is the choice of an appropriate baseline. Ambiguous baselines, such as a general “rest period” can reduce or change the sign of task-based BOLD signal change, due to the cognitive activity during the baseline condition (Stark and Squire 2001). A possibility to overcome the dependency of a baseline choice is to study task-state functional connectivity, which provides information about regional interactions during tasks and reconfigurations of functional networks (Gonzalez-Castillo and Bandettini 2018). To understand how the effects of artificial vestibular stimulation on the coordination of the BOLD signal across the brain in healthy subjects is also the foundation of understanding disease-related alterations in vestibular patients. Hence, the aim of the following study was twofold: (1) to determine parts of the parietal operculum uniquely associated with vestibular stimulation and estimate the nociceptive side-effects of GVS, and (2) to investigate changes in the network architecture using task-state functional connectivity of the entire cortical network during stimulation uniquely associated with vestibular perception. Therefore, we compared task activations and functional network architecture of galvanic vestibular (GVS) to galvanic nociceptive stimulation (GNS) using an identical setup and stimulation protocol in two experiments during high-resolution functional magnetic resonance imaging (fMRI). To our knowledge, whole-brain functional network changes during vestibular stimulation were not investigated so far, we thus followed a hypothesis-free approach correcting the fMRI signal for activation-induced connectivity estimate inflation (Cole et al. 2019).

## Methods

### Participants

Participants underwent either one or both of two independent GVS fMRI experiments with either unilateral vestibular stimulation (GVS) on each mastoid separately, or with galvanic nociceptive stimulation (GNS). Twenty-six (13 female, mean age 28.6 years, age range 19–44) healthy subjects without any previous history of neuro-otological disorders were included. Left-handed participants were excluded as defined by a score below + 60 for right-sided dominance using the Edinburgh handedness assessment. Participants gave their informed consent and were monetarily compensated for their participation. Ethical approval was given by the local ethical board of the University Hospital of Ludwig-Maximilians-Universität München in accordance with the 2013 revision of the Declaration of Helsinki.

### Tasks and procedure

The GVS experiment and the GNS experiment were carried out on separate days to exclude inter-stimulus interactions. GVS and GNS were applied via mastoidal electrodes and a custom-made, battery-powered GVS-generator outside the Faraday cage and carbon electrodes. Small LC filters tuned for resonance at 64 MHz and resistors (1 kΩ) were placed between the electrodes and connection cable to the stimulus generator in order to prevent radio frequency pickup and propagation by the wires. The generator and cables were identical as described in (Stephan et al. 2005).

Nociceptive stimulation was performed by placing one electrode on one lateral mastoid and the second electrode 1 cm inferior to it. Each subject underwent test-stimulations outside the scanner to find the ideal electrode positioning, to ensure that subjects perceived exclusively pain and no vestibular sensations. The GNS experiment was repeated in two separate pseudo-randomized sessions for the left and the right mastoid.

For the GVS experiment, one electrode was placed on the mastoid, the other one on the cervical vertebra C7. Stimulation was performed for the left and the right mastoid separately. To minimize side effects during GVS, 3 g lidocaine creme anaesthetic crème (Emla, Aspen Germany GmbH, Bad Oldesloe, Germany) was applied to the skin above the mastoid process behind each ear one hour before the GVS experiment (Ruehl et al. 2017).

**Fig. 1:**
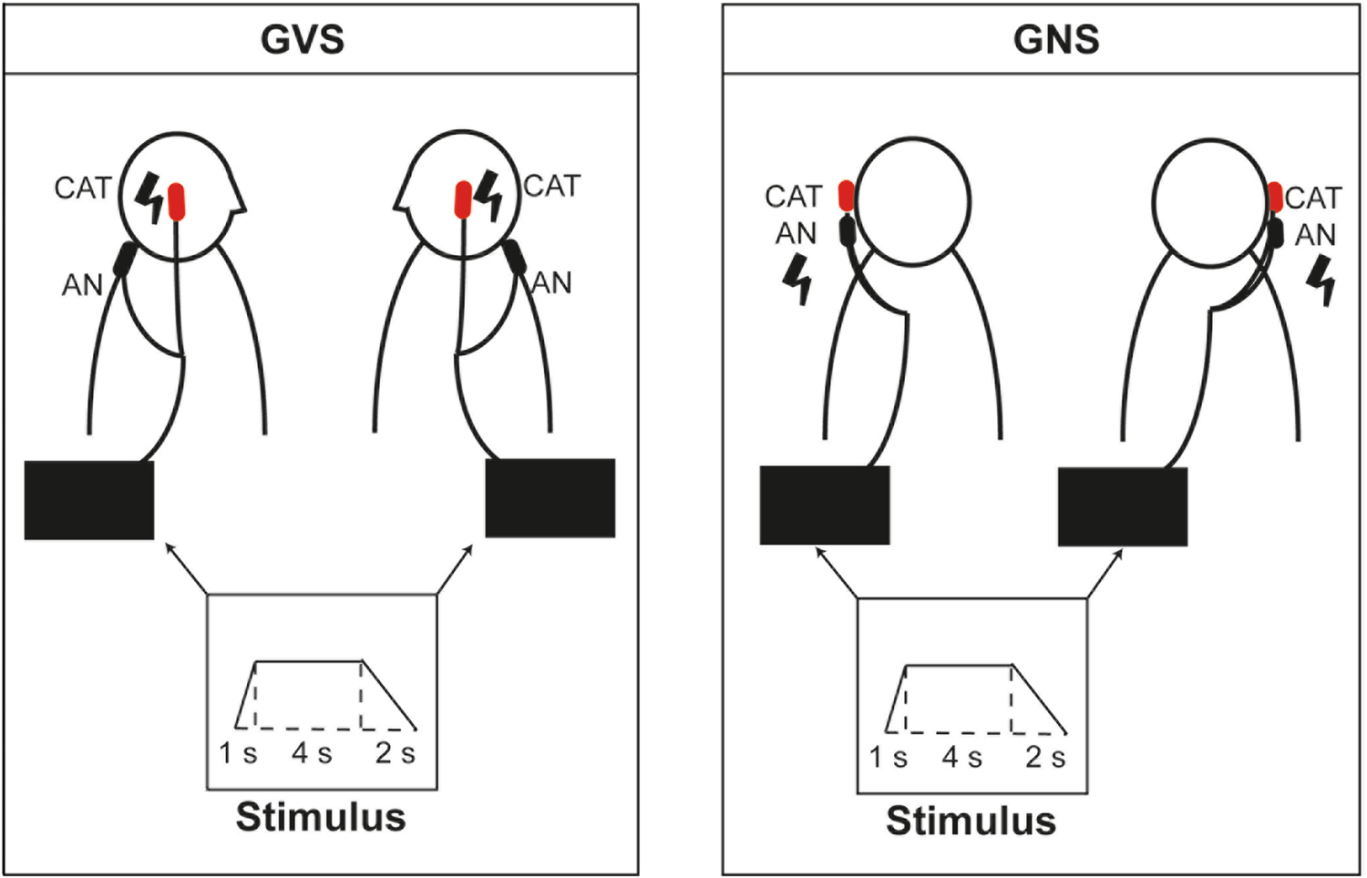
**Schematic drawing of both experimental setups**. During GVS-experiment (left section), either the left or the right mastoid was stimulated, and the anode was placed on the cervical vertebra C7. During the GNS-experiment (right) each mastoid was stimulated with the two electrodes placed on one mastoid. In both conditions, an identical step-wave stimulus was used

The stimulation protocol for both GVS and GNS was identical and consisted of a step waveform stimulus (1 s upward ramp, 4 s plateau and 2 s down) delivered either on the right or the left mastoid (see Fig. 1). Current intensities were adapted during both experiments to ensure a sufficient pain perception, which subjects were able to bear up during the entire session in the GNS session (mean stimulation strength 4 mA) and a pain-free vestibular perception during the GVS experiment (mean stimulation strength 3 mA). During both experiment, subjects were instructed to keep their eyes open and to look straight ahead at a white cross on a laminated black board on the scanner tunnel ceiling. The eyes open condition was chosen in order to guarantee an equal level of alertness during both the tasks and the resting state sequence. After each GVS and GNS session, subjects answered a standardized questionnaire including the rating of pain and vestibular sensations. During the GVS condition, participants expectedly reported egomotion, whereas during the GNS condition, no vestibular sensations were reported. The median pain rating given by the participants during the GNS condition was 4/10 (IQR = 2.25).

### MR acquisition

Data was collected with a clinical 3 T Magnetom Skyra scanner (Siemens, Erlangen, Germany) using a 64-channel head/neck coil. The resting-state (rs) session with eyes open prior to the tasks (7 min) and the task data (GNS and GVS) were acquired using a GE-EPI sequence (TR = 700 ms, TE = 33 ms, FA = 45°, multi-band factor = 6 with interleaved multi-band slice package order, voxel size = 2.5 mm isotropic, 54 slices, prescan normalised). A T1-weighted structural image was acquired using a MPRAGE Grappa sequence (TR = 2060 ms, TE = 2.17 ms, FA = 12°, voxel size: 0.75 mm isotropic, 256 slices) for DARTEL-based normalization in the subsequent preprocessing. All tasks were conducted in a block-design approach and consisted of identical stimulation protocols (block length for each stimulation 4.9 s, inter trial interval 9.1–16.1 s). High-resolution video-oculography was performed for during all sessions using an infrared VOG-unit (MRI-compatible camera, MRC systems, www.mrc-systems.de, frame rate 250 Hz) to ensure task adherence and attention during resting state and GVS/GNS. All participants received ear plugs and a gel capsule was attached on their right temple to ensure correct identification of side after preprocessing. During the experiment, participants were lying in the scanner in supine position, the head carefully fixed using an air-based cushion (Crania adult cap from Pearl Technology AG, Schlieren, Switzerland) to minimise head motion during the experiments.

### Analysis

#### General linear model (GLM) analysis

The task-based GLM-analysis was performed by means of SPM12 Version 7487 (https://www.fil.ion.ucl.ac.uk/spm/) and the SPM toolbox TFCE (r201 from 2020 to 04-21) in Matlab R2018a (9.4.0.949201 Update 6, MathWorks Inc., Natick, Massachusetts). The first 13 images (10 s) of each session were removed to account for T1-equilibration effects that go beyond the initial dummy scans removed by Siemens for fast fMRI protocols. The images were realigned to the first one of each scanning session and were then stereotactically normalized into the standard anatomical space defined by the Montreal Neurological Institute (MNI) template by means of the DARTEL algorithm including geodesic shooting using an existing MNI-template (http://nist.mni.mcgill.ca/?p=904) through the use of the CAT12 toolbox (version 1450) **(**Ashburner 2007). Therefore, the stereotactic coordinates in this paper refer to the MNI coordinate system. The normalized images were smoothed with a three-dimensional isotropic 4 mm Gaussian kernel and the realignment parameters and a high-pass filter (128 s) were integrated into the design matrix. The effect of the different stimulation conditions on regional BOLD responses was estimated according to the general linear model including the realignment parameters (Friston et al. 1995b). The conditions (GVS right, GVS left, GNS right, GNS left) were modelled as blocks.

Statistical parametric maps (SPMs) were generated on a voxel-by-voxel basis with a hemodynamic model of the stimulation periods present during the session (Friston et al. 1995a). To analyse differences in activations during both stimulations in general, we defined the contrasts to include the main effects for GVS applied on the left and right mastoid and for both the left and the right GNS experiments. These results are referred to as “vestibular stimulation” and “nociceptive stimulation” in the following sections.

Single subject t-contrasts were computed for each stimulation condition compared to the rest condition of each session and entered into a second-level statistical analysis to test for effects on a between-subject basis. Paired t-tests were performed between the GVS and GNS contrast, a conjunction analysis to test for areas significantly activated by both GSN and GVS and a correlation analysis including the pain scale and pain sensitivity questionnaire.

Statistical significance was determined using TFCE, with the default parameters after 10,000 permutations using a threshold of *p* < 0.05 corrected for multiple comparisons via false discovery rate (FDR) (Smith and Nichols 2009). When applicable and available, the cytoarchitectonic maps of the occipital and temporal lobe, the insular gyri and the parietal operculum were used to calculate the respective overlay of our results (Eickhoff et al. 2005). Results were localized and visualized using the anatomy toolbox (Eickhoff et al. 2005), the ANL atlas (Edlow et al. 2012) and MRIcroGL by Chris Rhorden (https://www.mccauslandcenter.sc.edu/mricrogl/).

#### Functional network analysis

After data quality control assessment via MRIQC (Esteban et al. 2017) to detect banding artefacts from multi-band imaging and excessive head movements, preprocessing for functional connectivity analysis was performed using fMRIPprep 1.2.5 (Esteban et al. 2019), based on Nipype 1.1.6 (Gorgolewski et al. 2011). T1 images were bias field corrected and skull stripped. Spatial normalisation was performed to the ICBM 152 Nonlinear Asymmetrical template version 2009c (Fonov et al. 2009) using nonlinear registration (see specifics in the online appendix) and brain tissue was segmented into cerebrospinal fluid, white matter and grey matter. BOLD images were registered to the normalised T1 image. Head motions parameters were estimated with six rotation and translation parameters. No slice timing correction was performed. BOLD times-series were resampled, corrected for head-motion and susceptibility distortions, and normalised to MNI152NLin2009cAsym space. Framewise displacement (FD) and DVARS were calculated and three region-wise global signals were extracted within the CSF, the WM, and the whole-brain masks. For detailed methods, see Online Appendix.

Fmriprep and MRIQC summary outputs were also used for quality control. Because functional connectivity data are particularly susceptible for motion, we used a strict inclusion criterion of a mean framewise displacement of FD > 0.2 as an output in MRQC in any run performed, or BOLD signal extinction in cortical brain areas after fmriprep preprocessing. For the within-group comparison applying these criteria resulted in a dataset of fifteen participants.

For further signal extraction and correction, CONN 18.b was used (Whitfield-Gabrieli and Nieto-Castanon 2012). Extraction was performed separately for the GVS and GNS data applying the same parameters. The reoriented and normalised functional data were used for signal extraction from 100 ROIs (7 Network parcellation), as defined by Schaefer et al. (2017). Data were despiked, detrended and filtered with a band-pass filter of 0.008–1 Hz to obtain a signal in the standard frequency range used for resting-state analysis. After filtering, regression was performed. For the stimulation sessions, we used a finite impulse response regressor to control for the influence of the mean event responses on functional connectivity values, as suggested by Cole et al. (2019). Further regressors included motion, CSF and WM signal as determined by fmriprep (raw signal as well as first-order derivative). High motion frames were also accounted for by creating a scrubbing regressor, which included all frames with a framewise displacement above 0.9 mm or BOLD signal changes above five standard deviations. Pearson correlation was calculated for the extracted and denoised signals and adjacency matrices were created for each participant and each condition. Each participant contributed to the analysis with six adjacency matrices in total: three from the GVS experiments (resting-state and GVS stimulation) and three from the GNS experiment (resting state, GNS stimulation left and GNS stimulation right). All further analysis steps were based on these correlation matrices.

General whole brain network changes associated with vestibular stimulation were determined using a within-participant design for the stimulation sessions (GVS and GNS) and the resting-state sessions from the two different experiments.

Two types of functional network analyses were conducted. The first analysis was performed using network-based statistics (NBS), which focuses on differences in individual connections within the network. The second analysis was focused on differences in modularity of the network, i.e. whether functionally related regions (i.e. groups of nodes) maintain or change their affiliation during different conditions. As a control, the two resting-state sessions of the different experiments within the same participant were compared, no changes in network architecture were expected there.

*Changes in network connectivity:* The NBS toolbox by Zalesky et al. (2010) was used to determine changes at the level of graph connections. In NBS, statistical tests are performed at every connection—only connections surpassing a primary threshold are further used to identify topological clusters. Considering the arbitrary nature of selecting the primary threshold, we used a range of primary thresholds (from 2 to 3.5 in steps of 0.3). For each component a FWER-corrected *p* value is determined with permutation testing at 10 000 permutations using the method of Freedman and Lane (1983). We only considered a component to be significant, if the *p* value was below 0.1 consistently across all primary thresholds tested. Both component extent and component intensity were investigated. Weak effects that include many connections tend to become significant with component extent, whereas testing for component intensity is better for detecting strong, focal connections.

*Changes in network modularity:* To determine how nodes differ in terms of their functional network participation during the GVS and the GNS sessions, i.e. whether nodes interacted with the same nodes throughout the conditions or whether they changed in terms of their interactions, a consensus modularity analysis as described in Castrillon et al. (2020) was conducted using custom made Matlab and R scripts (4.0.2 within RStudio 1.3.1056). The analysis was only marginally modified from Castrillon et al. (2020). For each participant in each of the four conditions, classification was performed using the Louvain algorithm with a gamma of 1.3 (i.e. larger than the default value of 1 to detect smaller modules) and no pre-defined module affiliation. The parameter for consensus modularity analysis was left at tau = 0.4 (Castrillon et al. 2020; Lancichinetti and Fortunato 2012). The result of this analysis was the classification consistency (*z*) and diversity (*h*) for each node in each of the four conditions (i.e. both resting-state sessions and both stimulation sessions (GVS and GNS). Classification consistency was based on the within-module degree z-score [a within-module version of degree centrality (Rubinov and Sporns 2010)], classification diversity was based on participation coefficient, a measure of diversity of intermodular connections of individual nodes. Functions from the Brain Connectivity Toolbox (Rubinov and Sporns 2010; Bullmore and Sporns 2009) were used to calculate these graph measures. To determine significant differences in classification consistency and diversity between the four conditions, Kruskal–Wallis tests were performed.

## Results

### Task-based GLM- analysis

#### Contrast main effects vestibular > nociceptive stimulation

**Fig. 2:**
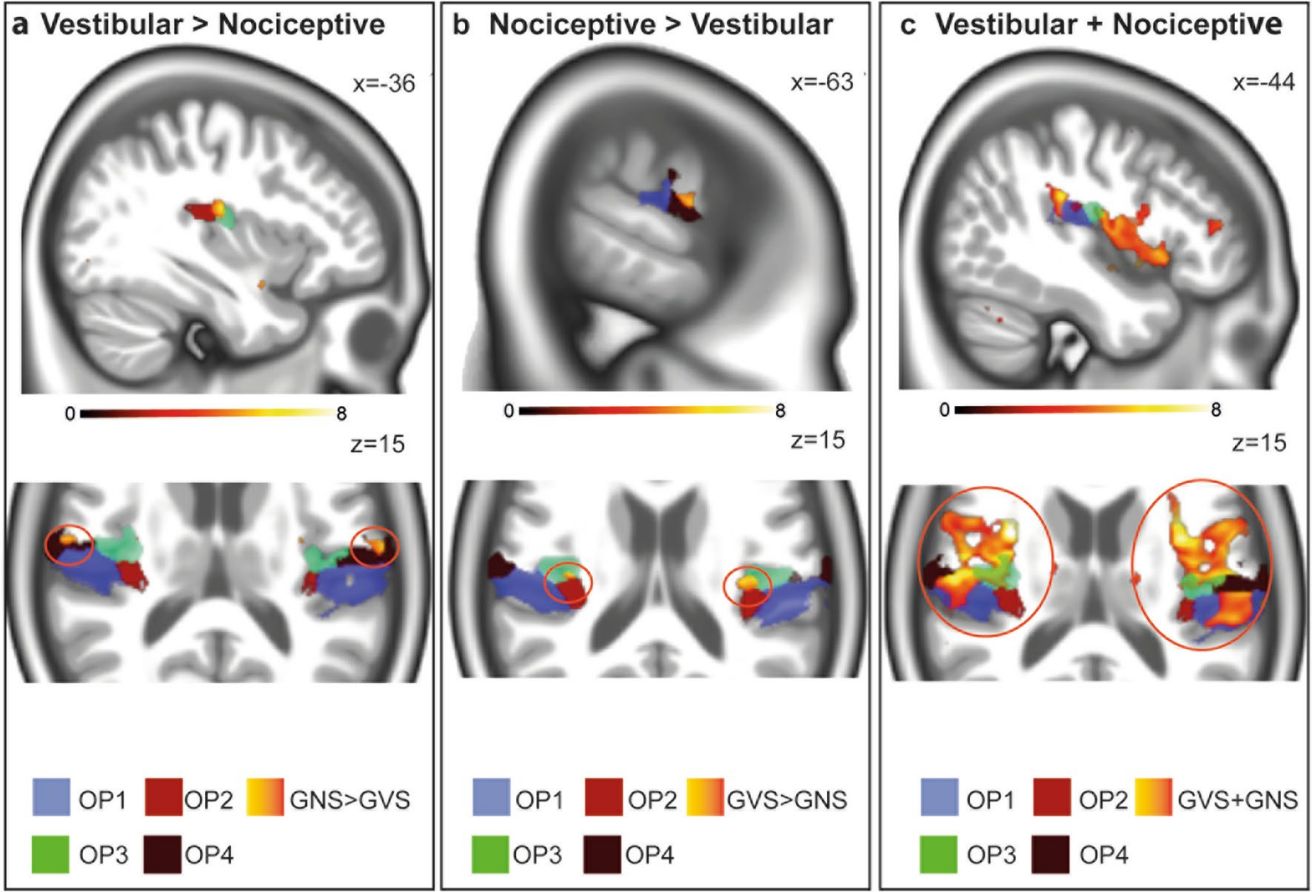
**Responses in the parietal operculum during the different tasks**. Nociceptive stimulation (yellow-orange) revealed stronger response in OP3 (green), as well as OP1 and OP4 (**a**) when contrasted with GVS. Vestibular stimulation (yellow-orange) gave signal increases in a cluster adjacent to OP4 (black) and adjacent to OP3 (not shown) compared to nociceptive stimulation (**b**). The conjunction analysis (yellow-orange, **c**) revealed common responses in area OP 1 (blue), OP3 (green) and OP4 black. Note that no conjunct activation of area OP2 was found. All activation maps were thresholded at *p* < 0.05, FDR TFCE

In the parietal operculum, GVS gave significant activations in the parietal operculum bilaterally in a cluster adjacent to OP3 and another cluster adjacent to OP4, not cytoarchitectonically localized (Fig. 2). Signal increases were also found in area CSv bilaterally, and in the cerebellum including the dorsal oculomotor vermis, lobule VIIIb, IX (uvula) and X (nodulus) of the vermis and right Crus II. Further increases were found in the right inferior frontal gyrus including area 44, the postcentral gyrus bilaterally, including area 4a and p, and the right putamen. In the inferior parietal lobule, signal increases were stronger in a cluster including area hIP3 bilaterally, corresponding to macaque area VIP. The detailed results for all contrasts can be found in supplemental table 1.

#### Contrast main effects nociceptive > vestibular stimulation

Nociceptive Stimulation revealed stronger activations of area OP1, OP3, OP4 and OP8 as well as parts of the parietal operculum adjacent to OP3 not cytoarchitectonically mapped so far. In the insular cortex, activations covered the anterior and mid- insular cortex including dysgranular area Id1. Further signal increases were found in area 44, the amygdala, the right hippocampus and cerebellar lobule VIIIA.

#### Conjunction analysis vestibular and nociceptive stimulation

The conjunction analysis revealed common peak response OP 1, 3, 4 and 8 bilaterally. Increased signal in the bilateral anterior insular cortex regions, consisting mostly of dysgranular areas in the midinsular/posterior insular cortex (Kurth et al. 2010), which are thought to process and mediate multisensory information (Benarroch 2019; Uddin 2015). Signal increases in the inferior parietal lobule extended bilaterally including area PFop, PFt. Further peaks were localized in area TE 1.2. (auditory cortex), as well as the putamen and in the anterior and posterior division of the cingulate gyrus bilaterally. In the primary somatosensory cortex, cluster were found in right cytoarchitectonic area 3b. In the cerebellum, bilateral lobule VIIb, lobule VI and Crus I were jointly activated in both tasks.

#### Main effects vestibular stimulation (left and right) > rest

**Fig. 3:**
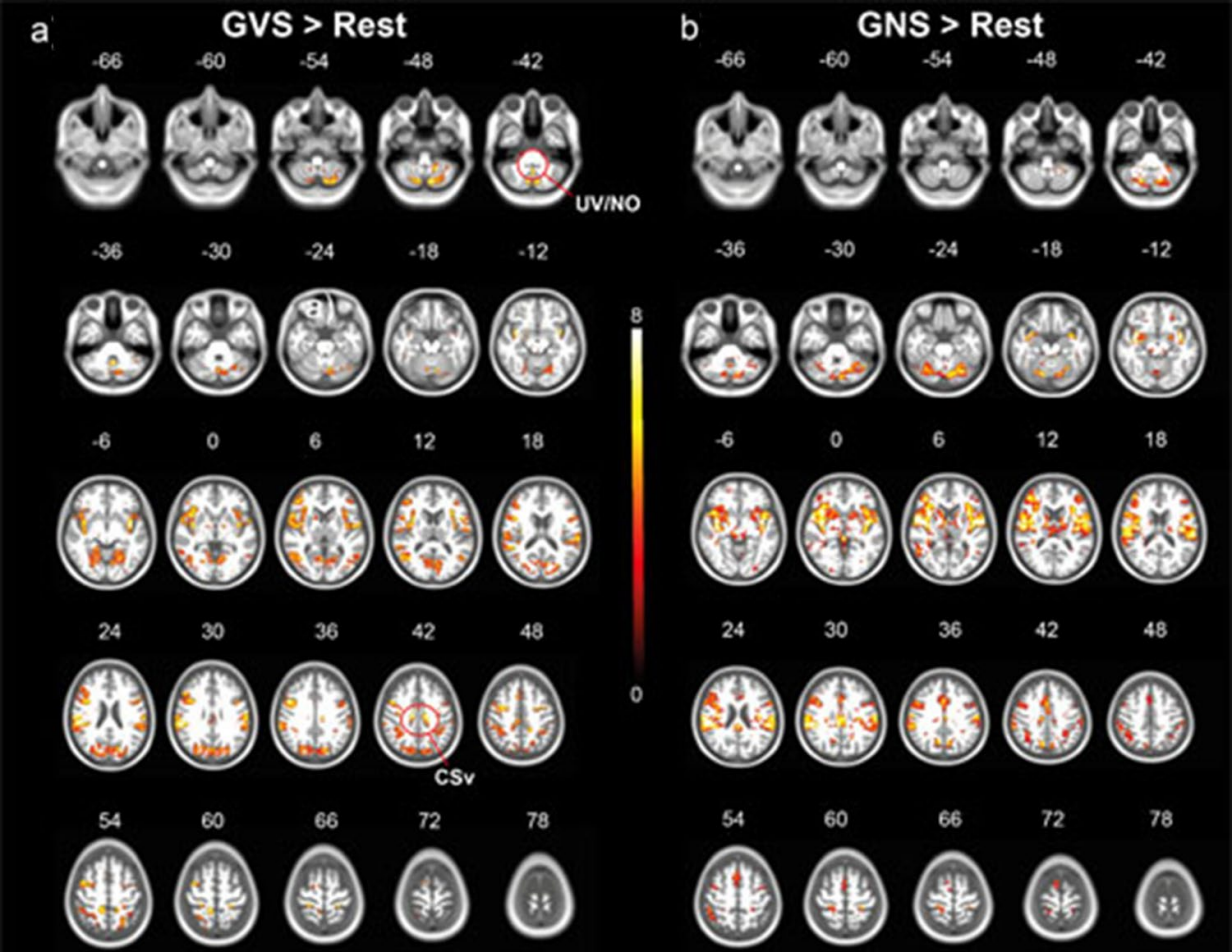
**Activation maps during vestibular and nociceptive stimulation**. Galvanic vestibular stimulation (red-yellow, **a**)) elicited responses in the parietal operculum, the anterior insula, area CSv, hMT and clusters extending from the postcentral gyrus bilaterally, the anterior cingulate gyrus and the inferior parietal lobule. In the cerebellum, signal increased in vestibulo-cerebellar core-regions (nodulus (NO), uvula (UV), flocculus, cerebellar tonsils) and in oculomotor-related regions (dorsal oculomotor vermis, interstitial nucleus of cajal). **b**) shows activation maps during nociceptive stimulation, including the parietal operculum (OP1, OP4), the anterior and posterior cingulate cortex, the anterior- and mid-insular cortex, the precuneus, the thalamus bilaterally, the prefrontal cortex, the inferior/posterior parietal cortex. In the brainstem, response were found in established areas related with pain processing (periaqueductal gray, pedunculopontine nucleus, nucleus gigantocellularis), whereas in the cerebellum response covered lobule VI, VIIb and left VIIIa. All activation maps were thresholded at *p* < 0.05, FDR TFCE

GVS elicited responses in the parietal operculum, including area OP1, 2, 3,4, area PFcm, parts of the anterior insular cortex, area CSv bilaterally, area hMT, a cluster extending from the postcentral gyrus including area 2, 3b, 4a, 6mc/SMA, the anterior cingulate gyrus, the inferior parietal lobule including area hIP1-3 (possible human correlate of macaque area VIP) (Fig. 3). Furthermore, activations were found in the putamen, the caudate and the thalamus bilaterally. On the infratentorial level, activations were found in vestibulo-cerebellar core regions (including the uvula, nodulus, flocculi,cerebellar tonsils) as well as the dorsal oculomotor vermis, Crus 1, 2, and lobule VI, VII, VIII bilaterally. In the brainstem, responses were found in the vestibular nuclei and in the mesencephalon, covering the interstitial nucleus of Cajal.

#### Main effects nociceptive stimulation (left and right) > rest

Nociceptive stimulation resulted in activations of the primary and secondary somatosensory cortex in the Rolandic operculum (OP1, OP4), the anterior and posterior cingulate cortex, the anterior- and mid-insular cortex,the precuneus, the thalamus bilaterally, the prefrontal cortex, the inferior/posterior parietal cortex (Fig. 3). On the infratentorial level, signal increases were found in the periaqueductal gray, the pedunculopontine nucleus, nucleus gigantocellularis and in the cerebellum bilaterally in lobule VI, VIIb and left VIIIa.

### Functional network changes related to vestibular stimulation

Functional connectivity differences within participants were analysed to determine a set of nodes (= *component*) with changes in functional connectivity associated with vestibular stimulation using the stimulation sessions (GVS and GNS) as well as the resting-state sessions from the two different experiments. By comparing the resting-state sessions from the different experiments within one subject, effects solely related to the different sessions could be disentangled.

#### Changes in network connectivity

To determine the connections associated with the change in experimental condition, the networks during GVS, GNS and rest were tested with network-based statistics. Seven primary thresholds were used for the NBS analysis. The contrast was only considered to be significant if the overall probability value was consistently below 0.1 across all thresholds tested. Differences were tested between the two stimulation datasets, the two resting-state (rs) datasets, and each stimulation dataset with its respective resting-state dataset. In each case, both extent and intensity were examined (see “Methods”).

**Fig. 4:**
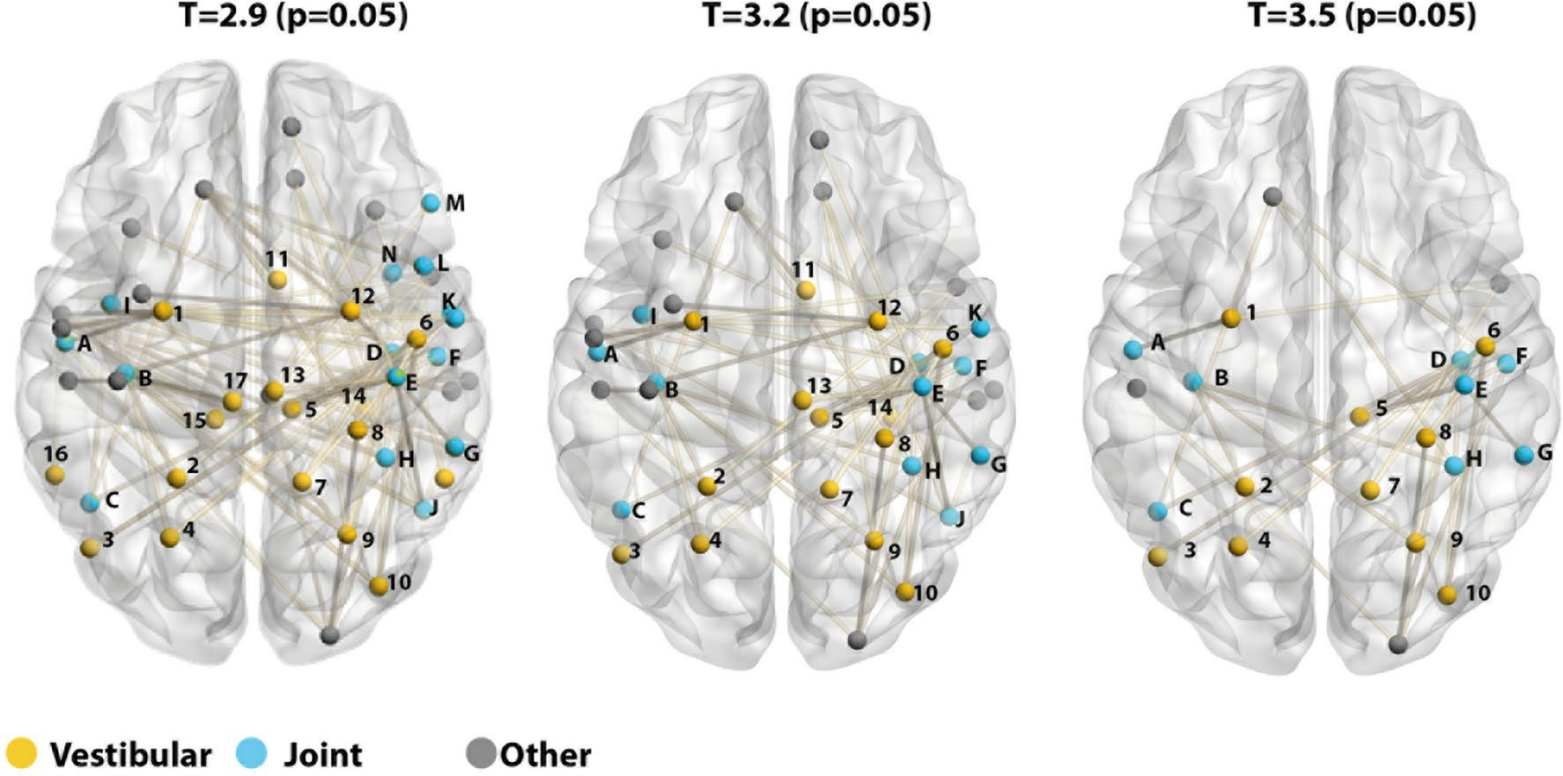
**Significant components for three incremental primary thresholds of the contrast GNS > GVS**. The results of the three thresholds are presented in incrementing order (left: *T* = 2.9; middle: *T* = 3.2; right: *T* = 3.5). Nodes were coloured according to the findings of the task-based analysis: yellow nodes were located in areas uniquely activated during GVS, blue nodes were located within regions jointly activated by GVS and GSN and grey nodes were located in other regions. Labels are shortened according to Schaefer et al. (2017) (legend is provided in supplemental Table 2)

In the comparison between GVS and GNS stimulation, we consistently identified a significant component associated with experimental condition. Specifically, vestibular stimulation was associated with a significant decrease of connectivity in a number of nodes located in regions, which were found to be associated with GVS in the task-based analysis (Fig. 4). Nodes were located both in regions uniquely activated by GVS (also including OP2 and CSV) as well as regions conjointly activated by both GVS and GNS. No other comparison resulted in significantly different components when testing for significant extent. The results were confirmed when testing for intensity instead of extent.

No differences were found between the two rs-fMRI sessions, confirming that the two imaging experiments did not change connections of the network and ruling out session effects. When testing for significant intensity, additional significant differences were found between GNS and its corresponding resting-state session. As the analysis was focussed on the vestibular system and not on the nociceptive condition per se, we did not follow up on these differences. Notably, no differences were found between GVS and rest. Overall, this suggests that changes in individual connections between nodes were driven by nociception and that vestibular stimulation had only a small effect on brain network architecture.

#### Changes in network modularity

**Fig. 5:**
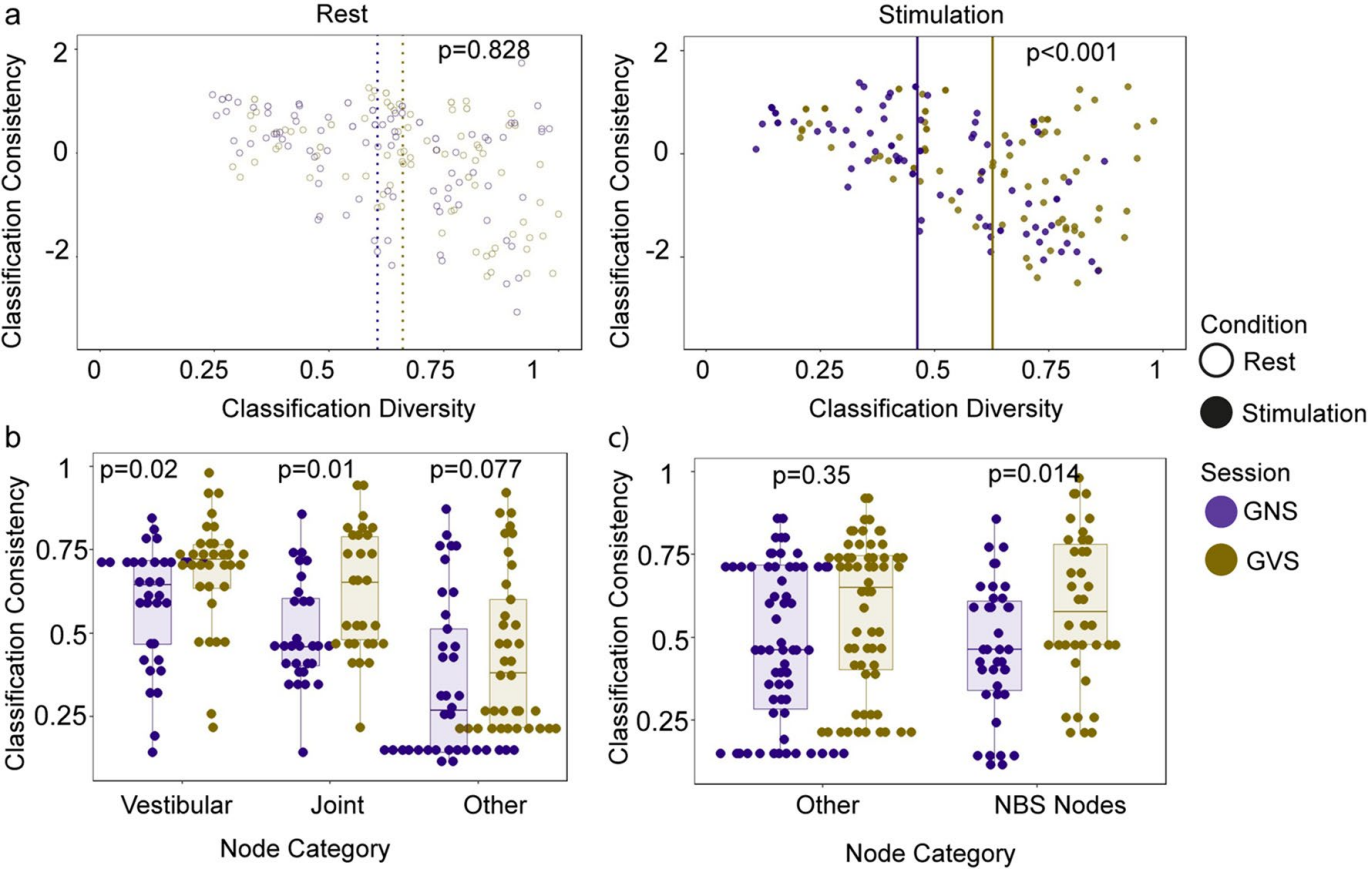
**Classification diversity and consistency**. **a** Classification diversity and classification consistency for all sessions and conditions. **b** Classification consistency of node categories derived from task-based analysis (Vestibular = nodes in regions significantly stronger activated by GVS, Joint = nodes in regions conjointly active during GVS and GNS, Other = all remaining nodes). **c** Classification consistency of node categories derived from NBS analysis (NBS Nodes = 38 nodes from the significant network found in the NBS analysis (using a threshold of *T* = 3.2, Other = remaining 62 nodes)

The NBS analysis showed that sets of connections are affected by the stimulation condition, with regions associated with vestibular processing being significantly less connected during GVS, when compared to GNS. To get a better understanding about the general network changes involved during the stimulation, we performed a modularity analysis (see “Methods”). Both classification consistency and classification diversity were calculated for each node in each condition. Classification consistency measures the extent of functional specialisation—a high-value means that the node is consistently classified as belonging to the same module. Conversely, classification diversity measures the proportion of nodes being classified into different modules and hence indicates that the node is well integrated into the network functionally. Low classification diversity means that a node is usually classified as belonging to the same module. Connectivity of such nodes is less dispersed across modules, while high classification diversity values suggest high dispersion of connectivity (Dwyer et al. 2014). Across all conditions, a significant difference was found for classification diversity (Kruskal–Wallis chi-squared = 29.172, df = 3, *p* value < 0.001) but not in classification consistency (Kruskal–Wallis chi-squared = 0.060, df = 3, *p* value = 0.996) (see Fig. 5A). This suggests that nodes within the brain were classified to variable modules across participants.

To determine the specific differences, Mann–Whitney–Wilcoxon tests were performed between all possible combinations using a Bonferroni correction (*α* = 0.5/6, adjusted *p* values are reported in the following). Classification diversity was significantly lower during GNS stimulation, when compared to the GVS stimulation (*U* = 6429, *p* = 0.003). Similarly, while classification diversity was significantly lower during GNS stimulation (median = 0.463), compared to the resting-state condition in the same scanning session (median = 0.661; *U* = 6570, *p* = 0.001), no difference was found when comparing the GVS stimulation (median = 0.637) to its corresponding resting-state condition (median = 0.606; *U* = 5830, *p* = 0.256). No session effect was found when comparing the two resting-state datasets from the two experiments (*U* = 5608, *p* = 0.828) (see Fig. 5A). These results suggest that cortical nodes become more selective in their interaction during nociceptive stimulation, whilst no reorganisation occurs during vestibular stimulation. To determine the contribution of different nodes to the differences in the stimulation conditions, we conducted two more post-hoc analyses.

First, we split the nodes into three groups, depending on whether they were located in regions that were activated uniquely by vestibular stimulation (“vestibular nodes”), jointly by vestibular and nociceptive stimulation (“joint nodes”) and all remaining nodes (“other nodes”). Indeed, both vestibular nodes (*W* = 356, *p* = 0.020) and joint nodes (*W* = 231, *p* = 0.010 had a higher classification diversity in the GVS condition. The remaining nodes did not differ in terms of their classification diversity (*W* = 478, *p* = 0.077) after Bonferroni correction (*α* = 0.5/3) (see Fig. 5B). Nodes located in regions associated with the stimulation conditions thus contributed to the changes in classification diversity more than the remaining nodes.

We also used a different categorisation and for this we split the nodes according to their membership of the significant NBS component found in the previous analysis. We thus tested whether the significantly decreased connections of these nodes during GVS (as found using the NBS analysis) is related with an increased classification diversity. For this, 38 nodes from the significant network found in the NBS analysis (using a threshold of *T* = 3.2, i.e. the nodes seen in the middle panel of Fig. 4) were included in the ‘NBS nodes’ groups, the remaining 62 nodes were included in the ‘Other’ group. As apparent in Fig. 5C, classification diversity significantly differed between the two stimulation periods but in both NBS nodes (*W* = 463, *p* = 0.014) as well as in all remaining nodes (*W* = 1447, *p* = 0.035) (adjusted *p* values after Bonferroni correction with *α* = 0.5/2). In this analysis, nodes thus contributed to the main finding, regardless whether they were part of the NBS component or not.

## Discussion

Our results highlight the importance of a somatosensory control stimulus when applying GVS in neuroimaging, as joint responses were found during nociceptive and vestibular stimulation in the parietal operculum of the secondary somatosensory cortex OP1 and OP4. The fact that no common responses were observed in area OP2 underlines its core role in vestibular processing. Contrasting both stimulation conditions, nociceptive stimulation led to larger responses in area OP3 and OP4, whereas vestibular stimulation gave stronger signal increases in parts of the parietal operculum so far not cytoarchitectonically localized adjacent to OP3 and OP4. Nociceptive stimulation was shown to have a significant impact on whole brain functional network connectivity, whereas vestibular stimulation did not.

### Multisensory processing during vestibular and nociceptive stimulation

Comparing the main effects of unilateral GVS and GNS stimulation revealed a common somatosensory pathway during both modalities. In the parietal operculum, the secondary somatosensory area OP1, areas OP3 and OP4 (BA43/40) were jointly active during both stimulation modalities, however, responses were stronger during nociceptive stimulation. OP4 is related to attention, stimulus discrimination, sensory-motor integration and action control (Eickhoff et al. 2010). This might explain our findings of a stronger involvement of OP4 during nociceptive stimulation, which requires an immediate reaction to the nociceptive stimulus. The stronger responses of OP3 to a nociceptive stimulus suits well to its association with encoding the somatosensory representation of the ear (Job et al. 2016, 2011). Our results further reveal signal increases during both stimulation modalities in the anterior and mid-insula, anterior and posterior parts of the cingulate gyrus and cluster in the inferior parietal lobule, which thus should not be considered as ‘unique’ vestibular responses. Taken together, these findings underline the importance of implementing a control stimulus to delineate vestibular responses and taking into account the multisensory side-effects of galvanic vestibular stimulation to correctly interpret vestibular stimulation results.

The operculum OP2 on the other hand was only responding to vestibular stimulation, but not to nociceptive (Fig. 2), which further hints at a central role in vestibular processing as proposed by zu Eulenburg et al. (2012). Furthermore, our findings substantiate the strong embedment of the cingulate sulcus visual (CSv) and parts of the inferior parietal lobule, including area PGp in the vestibular networks. These findings extend earlier studies, showing the importance of the CSv to visual and vestibular egomotion stimuli (Wall and Smith 2008; Smith et al. 2012). Evidence from both structural and functional connectivity suggest that it is connected with VIP and the parietal operculum (Smith et al. 2017). In the cerebellum, the nodulus, whose purkinje cells receive direct input from vestibular afferents (Cullen 2019; Yakusheva et al. 2010; Laurens et al. 2013; Goldberg et al. 2012), and the uvula gave stronger responses during GVS, as well as parts of Crus II. The responses in the dorsal oculomotor vermis (lobule VII) can be explained by the oculomotor responses elicited during vestibular, but during nociceptive stimulation.

### Effects on functional network architecture

Vestibular stimulation does not appear to have a significant impact on whole brain functional network connectivity. Despite the clear unique regional activation patterns associated with vestibular stimulation detected using a classic general linear model approach, the opposite was true when examining functional connectivity. We found that vestibular stimulation does not alter cortical network architecture: no significant differences in individual connections was found and modularity remained unchanged, when compared with resting state. Nociceptive stimulation on the other hand was associated with significant network changes compared with resting state. When compared directly with vestibular stimulation, it was associated with increased connectivity of regions, most of which were the same regions which also responded to galvanic and nociceptive stimulation in the task-based analysis.

This finding may be linked with the proposal by Klingner et al. (2016), who suggested that the amount of actual vestibular information (content) delivered to the cerebral cortex is relatively low compared to other (sensory) information transmitted. Another interpretation which we favour in light of our findings and the old age of the vestibular system within the family of senses is the continuous and ongoing processing of vestibular information in the awake human predominantly on a subconscious level. A recent work from our group demonstrated the robustness and low degree of vulnerability for the cortical vestibular system in a structural network approach. This robustness for the cortical vestibular system corresponds with the clinical experience with respect to cortical vestibular lesions (Raiser et al. 2020). There are no chronic vestibular symptoms (> 3 months) from supratentorial vestibular node injury (Babyar et al. 2015; Brandt and Dieterich 2017). To analyse differences in activations during both stimulations in general, we defined the contrasts to include the main effects for GVS applied on the left and right mastoid and for both the left and the right GNS experiments. These results are referred to as “vestibular stimulation” and “nociceptive stimulation” in the following sections. The lack of a global network reconfiguration in this study during a highly salient vestibular arousal in our opinion would argue for a stable and continuously active pre-existing network path for this kind (vestibular) of sensory input. Nociceptive processing seems to represent the exact opposite in this regard.

Overall, global network organisation and hence synchronisation of the brain regions did not seem to be changed at all by vestibular stimulation. Considering that changes in awareness or arousal seem to be one main underlying factor for modulation of brain synchronisation (Lurie et al. 2019), this finding is remarkable considering that the stimulation induces a strong vestibular sensation and elicits a distinct brain activity pattern. It is particularly noteworthy, that even when comparing classification diversity and consistency of the resting-state condition with the stimulation condition, no differences were found. This stability of brain synchronisation during vestibular sensation possibly reflects that vestibular processing occurs all the time in an awake state and is mostly subconsciously. Actual synchronisation effects during vestibular stimulation may be more subtle compared with nociceptive processing in the cortex.

## Conclusion

Our results reveal a common multisensory trunk during galvanic vestibular and nociceptive processing involving areas OP1, 3, 4 only excluding OP2 in the parietal operculum. Contrasting both stimulation modalities revealed stronger responses in parts of the parietal operculum, area CSv and the uvula, nodulus and Crus II in the cerebellum exclusively during vestibular stimulation. Our results underline the importance of a somatosensory control stimulus when using galvanic vestibular stimulation. Only nociceptive stimulation modulated the functional network, but vestibular stimulation did not lead to a change in global network properties for the respective cortical vestibular nodes. This may reflect the permanence and continuity of vestibular information processing on a subconscious level within an omnipresent network structure in awake and alert humans. It would explain why the vestibular sense did not end up on Aristotle’s list of essential senses. In a subsequent step, the contribution of subcortical vestibular regions should be analysed to determine whether the observed lack of network modulation is limited to cortical regions.

## Supplementary Information

Below is the link to the electronic supplementary material.

Supplementary file1 (DOCX 31 KB)

## Data Availability

Statistical group data and Matlab stimuli scripts are available upon reasonable request.
