# Supplementary material for: Delineating neural responses and functional connectivity changes during vestibular and nociceptive stimulation reveal the uniqueness of cortical vestibular processing
Source: Brain Struct Funct. 2021 Oct 5;227(3):779–91. doi: 10.1007/s00429-021-02394-6 (PMC8930960; doi:10.1007/s00429-021-02394-6)
Supplement: Supplementary file 1 — Supplementary file1 (DOCX 31 KB) [file 429_2021_2394_MOESM1_ESM.docx]

**Appendix**

- 1. Supplementary Methods

For anatomical data preprocessing, the T1-weighted (T1w) image was corrected for intensity non-uniformity (INU) using N4BiasFieldCorrection (Tustison et al. 2010, ANTs 2.2.0), and used as T1w-reference throughout the workflow. The T1w-reference was then skull-stripped using antsBrainExtraction.sh (ANTs 2.2.0), using OASIS as target template. Brain surfaces were reconstructed using recon-all (FreeSurfer 6.0.1, RRID:SCR_001847, Dale, Fischl, and Sereno 1999), and the brain mask estimated previously was refined with a custom variation of the method to reconcile ANTs-derived and FreeSurfer-derived segmentations of the cortical gray-matter of Mindboggle (RRID:SCR_002438, Klein et al. 2017). Spatial normalization to the ICBM 152 Nonlinear Asymmetrical template version 2009c (Fonov et al. 2009, RRID:SCR_008796) was performed through nonlinear registration with antsRegistration (ANTs 2.2.0, RRID:SCR_004757, Avants et al. 2008), using brain-extracted versions of both T1w volume and template. Brain tissue segmentation of cerebrospinal fluid (CSF), white-matter (WM) and grey-matter (GM) was performed on the brain-extracted T1w using fast (FSL 5.0.9, RRID:SCR_002823, Zhang, Brady, and Smith 2001).

For each of the BOLD runs found per subject (across all tasks and sessions), the following functional preprocessing was performed. First, a reference volume and its skull-stripped version were generated using a custom methodology of fMRIPrep. A deformation field to correct for susceptibility distortions was estimated based on fMRIPrep’s 2019s fieldmap-less approach. The deformation field is that resulting from co-registering the BOLD reference to the same-subject T1w-reference with its intensity inverted (Wang et al. 2017; Huntenburg 2014). Registration is performed with antsRegistration (ANTs 2.2.0), and the process regularized by constraining deformation to be nonzero only along the phase-encoding direction, and modulated with an average fieldmap template (Treiber et al. 2016). Based on the estimated susceptibility distortion, an unwarped BOLD reference was calculated for a more accurate co-registration with the anatomical reference. The BOLD reference was then co-registered to the T1w reference using bbregister (FreeSurfer) which implements boundary-based registration (Greve and Fischl 2009). Co-registration was configured with nine degrees of freedom to account for distortions remaining in the BOLD reference. Head-motion parameters with respect to the BOLD reference (transformation matrices, and six corresponding rotation and translation parameters) are estimated before any spatiotemporal filtering using mcflirt (FSL 5.0.9, Jenkinson et al. 2002). The BOLD time-series, were resampled to surfaces on the following spaces: fsaverage. The BOLD time-series were resampled onto their original, native space by applying a single, composite transform to correct for head-motion and susceptibility distortions. These resampled BOLD time-series will be referred to as preprocessed BOLD in original space, or just preprocessed BOLD. The BOLD time-series were resampled to MNI152NLin2009cAsym standard space, generating a preprocessed BOLD run in MNI152NLin2009cAsym space. First, a reference volume and its skull-stripped version were generated using a custom methodology of fMRIPrep. Several confounding time-series were calculated based on the preprocessed BOLD: framewise displacement (FD), DVARS and three region-wise global signals. FD and DVARS are calculated for each functional run, both using their implementations in Nipype (following the definitions by Power et al. 2014). The three global signals are extracted within the CSF, the WM, and the whole-brain masks. Additionally, a set of physiological regressors were extracted to allow for component-based noise correction (CompCor, Behzadi et al. 2007). Principal components are estimated after high-pass filtering the preprocessed BOLD time-series (using a discrete cosine filter with 128s cut-off) for the two CompCor variants: temporal (tCompCor) and anatomical (aCompCor). Six tCompCor components are then calculated from the top 5% variable voxels within a mask covering the subcortical regions. This subcortical mask is obtained by heavily eroding the brain mask, which ensures it does not include cortical GM regions. For aCompCor, six components are calculated within the intersection of the aforementioned mask and the union of CSF and WM masks calculated in T1w space, after their projection to the native space of each functional run (using the inverse BOLD-to-T1w transformation). The head-motion estimates calculated in the correction step were also placed within the corresponding confounds file. All resamplings can be performed with a single interpolation step by composing all the pertinent transformations (i.e. head-motion transform matrices, susceptibility distortion correction when available, and co-registrations to anatomical and template spaces). Gridded (volumetric) resamplings were performed using antsApplyTransforms (ANTs), configured with Lanczos interpolation to minimize the smoothing effects of other kernels (Lanczos 1964). Non-gridded (surface) resamplings were performed using mri_vol2surf (FreeSurfer).

Many internal operations of fMRIPrep use Nilearn 0.5.0 (Abraham et al. 2014, RRID:SCR_001362), mostly within the functional processing workflow. For more details of the pipeline, see the section corresponding to workflows in fMRIPrep\u2019s documentation.

# Supplementary Results

| **Table 1**  **Results of the cluster peaks (t-contrasts) for all conditions**  (TFCE, FDR corrected, p< 0.05) | | | | |
| --- | --- | --- | --- | --- |
| **T-contrast** | **Brain area** | **Cluster**  **size** | **t value** | **x, y, z** |
| **Conjunction GVS and GNS** |  |  |  |  |
| **Activations** | Parietal operculum area OP1 | 43443 | 10.45 | 53 -25 22 |
|  | Parietal operculum area OP4 |  | 10.02 | 52 1 6 |
|  | Anterior insula |  | 9.8 | 40 0 -1 |
|  | Parietal operculum area OP3 |  | 9.61 | 49 -2 9 |
|  | Anterior Insula |  | 9.38 | 41 -2 -9 |
|  | Area 44 | 30622 | 8.71 | -58 1 7 |
|  | Anterior insula |  | 8.54 | -40 2 -8 |
|  | Parietal operculum area OP4 |  | 8.46 | -52 -3 11 |
|  | Parietal operculum area OP8 |  | 8.37 | -34 12 11 |
|  | Cerebellar R lobule VI | 8002 | 6.08 | 29 -61 -20 |
|  | Cerebellar L crus II |  | 6.03 | -9 -79 -31 |
|  | Cerebellar R crus VIIb |  | 5.87 | 21 -71 -44 |
|  | Cerebellar L VIIb |  | 5.3 | -13 -70 -43 |
|  | Cerebellar R VIIb |  | 5.29 | 17 -71 -42 |
|  | Cingulate gyrus, anterior division | 3888 | 5.10 | 4 14 39 |
|  | Superior frontal gyrus |  | 5.01 | 6 20 53 |
|  | Cingulate gyrus, posterior division | 2361 | 5.87 | 3 -30 29 |
|  | Cerebellum R lobule VI | 2077 | 5.25 | 27 -65 -19 |
|  | Precuneus, **ar**ea hIP8 | 891 | 4.88 | 13 -70 41 |
|  | Precuneus, area 7A | 847 | 4.69 | -8 -67 43 |
|  | R Caudate | 758 | 5.09 | 16 -2 20 |
|  | Frontal pole | 662 | 4.14 | -42 43 13 |
|  | Postcentral gyrus , area 3b | 606 | 5.31 | 26 -31 69 |
|  |  |  | 4.05 | 26 -32 59 |
|  | R Thalamus | 405 | 4.93 | 13 -22 -6 |
|  | L Thalamus | 383 | 4.64 | -16 -11 13 |
|  | Postcentral gyrus | 362 | 5.88 | -26 -33 64 |
|  | Middle frontal gyrus | 356 | 4.37 | -41 2 59 |
|  | Mesencephalon | 195 | 4.31 | 10 -28 -9 |
|  | Cerebellum R Crus I | 111 | 3.64 | 46 -59 -28 |
|  | L caudate | 55 | 3.34 | -13 5 15 |
|  | Cerebellum L Crus I | 52 | 3.43 | 44 -59 -31 |
|  |  |  |  |  |
| **Nociceptive> vestibular** | Anterior insula | 330 | 7.32 | 37 2 -10 |
|  | Parietal operculum, area OP4 | 324 | 6.65 | 51 0 1 |
|  | Parietal operculum, area OP3 | 165 | 5.19 | -32 -18 20 |
|  | Insular cortex, area Id1 | 83 | 5.54 | 39 -4 -15 |
|  | Insular cortex | 42 | 5.6 | 40 -4 -5 |
|  | Insular cortex area Id1 | 40 | 5.95 | 38 -17 -7 |
|  | Parietal operculum, area OP8 | 36 | 5.16 | 32 11 8 |
|  | Anterior insula, amygdala | 33 | 4,81 | -34 4 -16 |
|  | Parietal operculum, area OP1 | 31 | 4.42 | 50 -14 15 |
|  | Area TE 1.2 | 32 | 4.77 | -57 -2 4 |
|  | Parietal operculum, area OP3 | 32 | 4.79 | 36 -15 18 |
| **Vestibular> nociceptive** | Parietal operculum, area OP4 | 2606 | 5.99 | -65 -8 13 |
|  | Precentral gyrus area 44 |  | 5.57 | -56 0 25 |
|  | Area 3a |  | 5.4 | -46 -10 27 |
|  | Area 3 b | 2188 | 6.19 | 58 -4 29 |
|  | Area 4a | 494 | 4.89 | 2 -30 55 |
|  | Parietal operculum, area OP3 | 264 | 5.42 | 36 -3 9 |
|  | Area CSv | 263 | 5.13 | 9 -13 51 |
|  | Area CSv | 240 | 4.83 | -14 -17 44 |
|  | Cerebellar vermis VIIb | 219 | 5.62 | -1 -67 -26 |
|  | Parietal operculum | 167 | 5.47 | -32 -7 17 |
|  | Cerebellar vermis VIIIb | 146 | 4.77 | 1 -61 -35 |
|  | Inferior parietal lobule, Area Pgp, hIP3 (VIP) | 79 | 5.03 | 43 -79 26 |
|  | Area 44 | 71 | 4.91 | 54 19 1 |
|  | Inferior parietal lobule, Area Pgp, hIP3 (VIP) | 49 | 4.25 | -42 -79 28 |
|  | Cerebellar vermis IX (Uvula) | 40 | 4.16 | 1 -52 -39 |
|  | Area 3a | 37 | 5.53 | -12 -32 60 |
|  | Right putamen | 36 | 4.46 | 26 -1 13 |
|  | Crus II | 28 | 4.10 | 16 -78 -41 |
|  | Area 3a | 28 | 4.10 | 16 -78 -41 |
|  | Cerebellar vermis X (Nodulus) | 25 | 4.44 | 2 -49 -30 |
| **Vestibular>rest** | Area PFcm | 143605 | 9.91 | 55 -31 30 |
|  | Area 44 |  | 9.85 | 46 7 37 |
|  | Anterior Insular Cortex |  | 9.58 | 37 1 -11 |
|  | Area PFop |  | 9.35 | 60 -18 23 |
|  | Cerebellum L VI |  | 9.03 | -9 -66 -21 |
|  | Parietal operculum |  | 8.77 | 47, -27, 24 |
|  | Anterior insula cortex | 28334 | 9.62 | -39 0 -7 |
|  | Area PFop |  | 8.23 | -62 -20 26 |
|  | Parietal operculum |  | 7.03 | -49 -6 6 |
|  | CSv | 6985 | 9.24 | 11 -14 43 |
|  | Area 3b |  | 8.49 | 14 -42 59 |
|  | Area 2 |  | 8.01 | 27 -34 63 |
|  | Area 4a |  | 5.54 | 4 -24 50 |
|  | Area 6mc/SMA |  | 4.69 | 4 -7 41 |
|  | Anterior cingulate cortex |  | 4.41 | 1 -6 46 |
|  | Area 4a | 5205 | 8.45 | -23 -35 68 |
|  | Area 3 b |  | 7.19 | -24 -35 60 |
|  | Area hIP2 |  | 5.24 | -49 -36 44 |
|  | Superior parietal lobule, Area 7PC |  | 5.1 | -34 -43 49 |
|  | Postcentral gyrus, area 3a |  | 5.08 | -27 -34 52 |
|  | Area hIP1 |  | 4.98 | -36 -39 42 |
|  | Area hIP3 |  | 4.72 | -42 -42 47 |
|  | Area 3a |  | 4.37 | -20 -31 55 |
|  | CSv | 3659 | 9.58 | -12 -17 45 |
|  | Area 5M |  | 7.63 | -15 -39 51 |
|  | Precentral gyrus |  | 7.12 | -17 -24 40 |
|  | Area 5Ci |  | 4.27 | -19 -36 43 |
|  | Lateral occipital cortex | 3540 | 5.71 | -39 -63 8 |
|  | Middle temporal gyrus |  | 5.97 | -45 -62 11 |
|  | Middle frontal gyrus | 2395 | 5.94 | -40 -1 49 |
|  | Superior frontal gyrus, area 6d3 |  | 5.67 | -24 -2 47 |
|  | Frontal Pole | 1891 | 5.10 | -39 37 9 |
|  | Cerebellum L VIIIb, IX (Tonsil) | 1549 | 6.35 | -118 |
|  | Area 6mr, preSMA | 1303 | 6.48 | 9 15 52 |
|  | Lateral occipital cortex | 938 | 4.81 | -30 -79 10 |
|  | Cerebellum R IX (Cerebellar tonsil) | 416 | 4.86 | 12 -51 -52 |
|  | Cerebellum R VIIIb |  | 3.54 | 16 -55 -45 |
|  | R Caudate | 399 | 4.23 | 13 10 8 |
|  | L Thalamus | 387 | 5.03 | -12 -6 1 |
|  | R Thalamus | 326 | 4.77 | 11 -5 2 |
|  | Area Fo3 | 322 | 5.92 | 24 39 -12 |
|  | Area FG4 | 295 | 4.76 | 48 -47 -19 |
|  | Area 6mr/pre SMA | 268 | 4.54 | 3 3 58 |
|  | L Caudate | 263 | 4.30 | -13 11 5 |
|  | R Thalamus | 221 | 4.22 | 12 -20 -5 |
|  | Cerebellum L Crus I | 195 | 4.44 | -39 -53 -35 |
|  | R Amygdala | 150 | 5.37 | 19 -2 -12 |
|  | Parietal operculum | 147 | 4.83 | -33 -16 17 |
|  | Cerebellum L VI | 132 | 4.99 | -39 -42 -36 |
|  | Mesencephalon | 119 | 4.96 | 7 -25 -20 |
|  | R Putamen | 104 | 4.58 | 25 4 -3 |
|  | Mesencephalon | 81 | 4.64 | 10 -24 -11 |
|  | R Caudate | 76 | 4.32 | 9 7 1 |
|  | Mesencephalon | 68 | 4.72 | -2 -30 -12 |
|  | Vestibular nuclei | 61 | 4.05 | -4 -40 -36 |
| **Nociceptive>rest** | Area 44 | 241748 | 16.92 | 37 2 -10 |
|  | Mid-insular cortex |  | 13.61 | -48 |
|  | Central opercular cortex |  | 13.00 | 39 -2 -2 |
|  | Area OP8 |  | 12.4 | 32 12 9 |
|  | Area PFcm |  | 11.9 | 46 -28 23 |
|  | Mesencephalon, Peduncolopontine nucleus |  | 11.1 | 9 -23 -16 |
|  | Precuneus | 3080 | 7.10 | 9 -68 39 |
|  | Frontal pole | 3063 | 4.82 | -43 44 12 |
|  | Occipital fusiform gyrus, Area FG1 | 2977 | 4.88 | -31 -75 -1 |
|  | Postcentral gyrus | 2299 | 5.35 | 23 -34 66 |
|  | Area 3b | 1506 | 6.53 | -26 -34 64 |
|  | Middle frontal gyrus | 1133 | 4.57 | -33 -8 41 |
|  | Occipital fusiform gyrus | 596 | 4.47 | 23 -86 -1 |
|  | Frontal pole | 541 | 4.56 | -25 34 -9 |
|  | Area FG4 | 520 | 5.23 | -110 |
|  | Pons, nucleus gigantocellularis | 254 | 5.17 | 11 -40 -42 |
|  | Occipital pole hOc3v | 236 | 3.89 | -16 -93 -7 |
|  | Lateral occipital cortex | 227 | 4.72 | 34 -79 -2 |
|  | Frontal pole | 159 | 3.95 | -26 40 17 |
|  | Pons, locus coeruleus | 140 | 4.16 | 4 -35 -28 |
|  | Cerebellum R IV | 137 | 3.38 | 21 -40 -33 |
|  | R Thalamus | 129 | 3.33 | -8 -32 15 |
|  | Cerebellum R VIII A | 127 | 3.54 | 25 -50 -41 |
|  | Cerebellum R lobule V | 127 | 3.54 | 25 -50 -41 |
|  | L Thalamus | 98 | 3.71 | -21 -34 0 |
|  | R Thalamus, hippocampus | 90 | 3.13 | 21 -33 -2 |

Table 2: Supplemental legend for figure 3

| Nodes of the joint network | |
| --- | --- |
| A | R SM3 Area |
| B | R SM2 OP2 |
| C | R DA p1 hMST |
| D | SM2 |
| E | L SM6 |
| F | L SM1 OP2 |
| G | SVA TempOccPar1 PFArea7 |
| H | DAp3 IPS3 |
| I | SVA FrOperIns1 |
| J | R Vis3 hMST |
| K | R SM4 Area 6 premotor |
| L | DA PrCv1_Area6Premotor |
| M | D PFCv2 |
| N | SVA FrOperIns1 |

| Nodes of the vestibular network | |
| --- | --- |
| 1 | L DA2 FEF |
| 2 | L DA p6 |
| 3 | L Vis 7 |
| 4 | L DA p3 |
| 5 | R SVA Med1 CSv |
| 6 | R SM5 |
| 7 | R DA p5 |
| 8 | R SM7 Area 2v/ 3aV |
| 9 | DA p4 |
| 10 | R Vis 7 |
| 11 | R SVA Med2 Periarcuate SMA |
| 12 | R DA FEF1 Area 6 Premotor |
| 13 | R SM8 Periarcuate SMA |
| 14 | Vis1 |
| 15 | SVA Med2 CSv |
| 16 | D Par1 |
| 17 | SM6 area2 area3aV |

## Supplementary References

Abraham, Alexandre, Fabian Pedregosa, Michael Eickenberg, Philippe Gervais, Andreas Mueller, Jean Kossaifi, Alexandre Gramfort, Bertrand Thirion, and Gael Varoquaux. 2014. “Machine Learning for Neuroimaging with Scikit-Learn.” Frontiers in Neuroinformatics 8. https://doi.org/10.3389/fninf.2014.00014.

Avants, B.B., C.L. Epstein, M. Grossman, and J.C. Gee. 2008. “Symmetric Diffeomorphic Image Registration with Cross-Correlation: Evaluating Automated Labeling of Elderly and Neurodegenerative Brain.” Medical Image Analysis 12 (1): 26–41. https://doi.org/10.1016/j.media.2007.06.004.

Behzadi, Yashar, Khaled Restom, Joy Liau, and Thomas T. Liu. 2007. “A Component Based Noise Correction Method (CompCor) for BOLD and Perfusion Based fMRI.” NeuroImage 37 (1): 90–101. https://doi.org/10.1016/j.neuroimage.2007.04.042.

Dale, Anders M., Bruce Fischl, and Martin I. Sereno. 1999. “Cortical Surface-Based Analysis: I. Segmentation and Surface Reconstruction.” NeuroImage 9 (2): 179–94. https://doi.org/10.1006/nimg.1998.0395.

Esteban, Oscar, Ross Blair, Christopher J. Markiewicz, Shoshana L. Berleant, Craig Moodie, Feilong Ma, Ayse Ilkay Isik, et al. 2018. “FMRIPrep 1.2.5.” Software. Zenodo. https://doi.org/10.5281/zenodo.852659.

Esteban, Oscar, Christopher Markiewicz, Ross W Blair, Craig Moodie, Ayse Ilkay Isik, Asier Erramuzpe Aliaga, James Kent, et al. 2018. “FMRIPrep: A Robust Preprocessing Pipeline for Functional MRI.” bioRxiv. https://doi.org/10.1101/306951.

Fonov, VS, AC Evans, RC McKinstry, CR Almli, and DL Collins. 2009. “Unbiased Nonlinear Average Age-Appropriate Brain Templates from Birth to Adulthood.” NeuroImage, Organization for human brain mapping 2009 annual meeting, 47, Supplement 1: S102. https://doi.org/10.1016/S1053-8119(09)70884-5.

Gorgolewski, K., C. D. Burns, C. Madison, D. Clark, Y. O. Halchenko, M. L. Waskom, and S. Ghosh. 2011. “Nipype: A Flexible, Lightweight and Extensible Neuroimaging Data Processing Framework in Python.” Frontiers in Neuroinformatics 5: 13. https://doi.org/10.3389/fninf.2011.00013.

Gorgolewski, Krzysztof J., Oscar Esteban, Christopher J. Markiewicz, Erik Ziegler, David Gage Ellis, Michael Philipp Notter, Dorota Jarecka, et al. 2018. “Nipype.” Software. Zenodo. https://doi.org/10.5281/zenodo.596855.

Greve, Douglas N, and Bruce Fischl. 2009. “Accurate and Robust Brain Image Alignment Using Boundary-Based Registration.” NeuroImage 48 (1): 63–72. https://doi.org/10.1016/j.neuroimage.2009.06.060.

Huntenburg, Julia M. 2014. “Evaluating Nonlinear Coregistration of BOLD EPI and T1w Images.” Master’s Thesis, Berlin: Freie Universität. http://hdl.handle.net/11858/00-001M-0000-002B-1CB5-A.

Jenkinson, Mark, Peter Bannister, Michael Brady, and Stephen Smith. 2002. “Improved Optimization for the Robust and Accurate Linear Registration and Motion Correction of Brain Images.” NeuroImage 17 (2): 825–41. https://doi.org/10.1006/nimg.2002.1132.

Klein, Arno, Satrajit S. Ghosh, Forrest S. Bao, Joachim Giard, Yrjö Häme, Eliezer Stavsky, Noah Lee, et al. 2017. “Mindboggling Morphometry of Human Brains.” PLOS Computational Biology 13 (2): e1005350. https://doi.org/10.1371/journal.pcbi.1005350.

Lanczos, C. 1964. “Evaluation of Noisy Data.” Journal of the Society for Industrial and Applied Mathematics Series B Numerical Analysis 1 (1): 76–85. https://doi.org/10.1137/0701007.

Power, Jonathan D., Anish Mitra, Timothy O. Laumann, Abraham Z. Snyder, Bradley L. Schlaggar, and Steven E. Petersen. 2014. “Methods to Detect, Characterize, and Remove Motion Artifact in Resting State fMRI.” NeuroImage 84 (Supplement C): 320–41. https://doi.org/10.1016/j.neuroimage.2013.08.048.

Treiber, Jeffrey Mark, Nathan S. White, Tyler Christian Steed, Hauke Bartsch, Dominic Holland, Nikdokht Farid, Carrie R. McDonald, Bob S. Carter, Anders Martin Dale, and Clark C. Chen. 2016. “Characterization and Correction of Geometric Distortions in 814 Diffusion Weighted Images.” PLOS ONE 11 (3): e0152472. https://doi.org/10.1371/journal.pone.0152472.

Tustison, N. J., B. B. Avants, P. A. Cook, Y. Zheng, A. Egan, P. A. Yushkevich, and J. C. Gee. 2010. “N4ITK: Improved N3 Bias Correction.” IEEE Transactions on Medical Imaging 29 (6): 1310–20. https://doi.org/10.1109/TMI.2010.2046908.

Wang, Sijia, Daniel J. Peterson, J. C. Gatenby, Wenbin Li, Thomas J. Grabowski, and Tara M. Madhyastha. 2017. “Evaluation of Field Map and Nonlinear Registration Methods for Correction of Susceptibility Artifacts in Diffusion MRI.” Frontiers in Neuroinformatics 11. https://doi.org/10.3389/fninf.2017.00017.

Zhang, Y., M. Brady, and S. Smith. 2001. “Segmentation of Brain MR Images Through a Hidden Markov Random Field Model and the Expectation-Maximization Algorithm.” IEEE Transactions on Medical Imaging 20 (1): 45–57. https://doi.org/10.1109/42.906424.
